# “Out-of-Hospital and with Qualified Exercise Professionals”: Keys to the CORTEX-SP Physical Exercise Program According to the Experience of the Participants

**DOI:** 10.3390/ejihpe13090125

**Published:** 2023-09-05

**Authors:** Mikel Tous-Espelosin, Uxue Fernandez-Lasa, Estibaliz Romaratezabala

**Affiliations:** 1 GIzartea, Kirola eta Ariketa Fisikoa Ikerkuntza Taldea (GIKAFIT), Society, Sports, and Physical Exercise Research Group, Department of Physical Education and Sport, Faculty of Education and Sport-Physical Activity and Sport Sciences Section, University of the Basque Country (UPV/EHU), 01007 Vitoria-Gasteiz, Spain; uxue.fernandez@ehu.eus (U.F.-L.); estibaliz.romaratezabala@ehu.eus (E.R.); 2 Physical Activity, Exercise, and Health Group, Bioaraba Health Research Institute, 01007 Vitoria-Gasteiz, Spain

**Keywords:** schizophrenia, physical exercise, program, benefits, qualitative

## Abstract

Physical exercise programs are useful and necessary for the treatment of schizophrenia. The aim of this study was to assess the experiences of participants with schizophrenia in an out-of-hospital exercise program designed and supervised by qualified exercise professionals. Thirty-five individuals with schizophrenia from the intervention group of the CORTEX-SP study were interviewed. The interviews were transcribed verbatim and content analysis was performed using inductive coding. Two main categories emerged: the importance of the program being conducted out-of-hospital, and the individuals responsible for the program being qualified exercise professionals. The participants highlighted the importance of conducting the program outside the psychiatric center since it gave them greater satisfaction. They perceived greater seriousness and a greater number of resources and felt encouraged to repeat the program or prolong it. The success of the program, in addition to the space, was due to the personnel in charge of the program, i.e., the qualified exercise professionals, and the fact that the activities were designed and supervised. Participants emphasized the qualifications of the exercise professionals, key for this type of program, their social skills and the level of involvement with participants and their follow-up.

## 1. Introduction

Schizophrenia (SP) is the most common psychotic mental disorder [1] and affects approximately 1% of the world population for life [2,3]. The diagnostic criteria for the disease include three main groups of symptoms: (a) positive symptoms, manifested in the form of hallucinations, delusions, and thought disorders that often lead to aggressive and risky behavior; (b) negative symptoms, including loss of functions such as speech disturbances, personal isolation, deprivation of emotions and feelings of pleasure, and abolishment; and (c) cognitive dysfunction, which causes deficits in attention, concentration, reasoning, and memory, as well as increased information-processing time [4,5]. These symptoms make individuals with SP have a personal perception, or that of others towards them, of a non-normal person. This fact leads to numerous barriers and limitations for the participation and inclusion of these individuals in society [2,6,7], such as feeling discriminated against [8], having difficulties in being connected with the environment, and achieving a sense of belonging [9].

For this reason, the social insertion of individuals with SP, among other factors, is based on the control of the psychotic symptoms of the disease [10] and, to that end, the psychotherapeutic and pharmacological treatments play a fundamental role [11]. Despite the fact that these treatments improve the positive symptoms of the disease [12], there seems to be a lack of effective treatments for the negative symptoms, including cognitive dysfunction [13]. In this sense, at present, physical exercise programs are presented as a non-pharmacological resource that is more than interesting and useful in the prevention and treatment of cognitive dysfunction. Further, these programs are taken into consideration for the positive symptoms caused by SP [11,14,15], thus helping improve the quality of life of individuals affected by this pathology [16].

However, physical exercise programs for this population face a double challenge. Individuals with SP, in comparison to individuals without the disease, have worse cardiorespiratory fitness and ventilatory capacity, medium to high heart rates, worse physical, physiological, and biochemical profile, and overweight/obesity [17]. Therefore, due to these conditions, performing physical exercise becomes a difficult task for these individuals [18]. In turn, these characteristics, together with the positive and negative symptoms caused by the disease, make it difficult to adhere to physical exercise programs [2]. In this sense, on the one hand, it is necessary that both the healthcare personnel and the closest family members [19] encourage and motivate individuals with SP to participate in physical exercise programs [7,18]. On the other hand, it is necessary to consider the importance of these programs being designed and supervised by exercise professionals, such as qualified exercise professionals, given that better results and lower dropout rates are obtained in this way [20].

Psychiatric hospitals provide a close and favorable environment for the initiation of physical exercise, offering a wide variety of exercise programs to their hospitalized patients [21]. However, less than half of patients participate in such programs, usually due to a lack of motivation [18] and poor physical condition [22]. Furthermore, once they are discharged from the hospital, they are no longer able to participate in these inpatient programs [21] and find it difficult to maintain physical exercise habits, either due to lack of out-of-hospital programs, lack of family support or health, the personal economy or the social context [19]. On the contrary, out-of-hospital exercise programs have been found to have higher acceptance rates, as well as better participation rates [23], and lower dropout rates. Finally, some studies concluded that the implementation of exercise programs designed and supervised by exercise professionals is more beneficial and positive for participants [18,20]. Therefore, the aim of the present study was to assess the meaning that participants with SP gave to an out-of-hospital exercise program designed and supervised by qualified exercise professionals.

## 2. Materials and Methods

This work is a part of a larger project in which different measurements were taken before and after the implementation of the program to evaluate clinical, psychiatric, psychological, physical, health, and social aspects. The qualitative methodology was used in the present study, specifically, the interview technique [24], which helps open the door to the universe of meanings [25] and allows the researchers to guide the areas of inquiry, leaving space so that the participants offer new meanings related to the topics [26]. Between February and March 2021, thirty-five semi-structured interviews (twenty-six men and nine women) were conducted with individuals from a psychiatric hospital and from the Mental Health Network of the Basque Country (Spain) that had been diagnosed with SP and belonged to the intervention group using physical exercise in the CORTEX-SP study [17,27]. In addition, two relatives participated as companions in two of the interviews. The interviews, with an average duration of forty-five minutes, were conducted by two members of the research team with the following structure: introduction and welcome; authorization obtained for audio recording; interview; and farewell.

All those individuals who were participating in the CORTEX-SP intervention group at the time of the interviews were interviewed. The inclusion criteria for participating in the study were being a participant in the physical exercise program of the CORTEX-SP study and being of legal age. All the interviewees voluntarily participated in the present study and signed the appropriate informed consent form. The study was approved by the local Research Ethics Committee (PI2017044).

Once the interviews were completed, they were transcribed verbatim and a content analysis was performed reading and analyzing the data obtained [28]. The data were coded in an inductive way and organized by thematic blocks [29] complying with the reliability and consistency criteria [30,31], to later group them into categories [32] (Table 1). This coding was performed by two members of the research team independently. Subsequently, the findings were used for triangulation between the researchers [24]. The content analysis was performed using the Nvivo9 software (QSR International).

## 3. Result

### 3.1. Out-of-Hospital Program

#### 3.1.1. Much Better Out-of-Hospital

Several participants expressed the importance of the program being out-of-hospital. In this way, the meaning attributed to it also differed and it was an opportunity to escape from routine, as stated by a participant: “it is also a way of escaping from the psychiatric hospital, which makes us crazier than we are” (William).

Changing the space brought the participants more benefits, since some expressed that changing scenery was related to getting away from the negative feelings associated with the psychiatric hospital: “you are not in the same place, because the psychiatric ward is a bit sad” (William).

Along the same line, the level of participation was related to the fact that the program was being conducted out of the psychiatric hospital. Some participants stated that their participation was conditional on the intervention being performed outside the psychiatric center, because they did not want to return to the past, as Isabella explained: “no, less in the psychiatric hospital, I would not have gone, no. I don’t like the psychiatric hospital. I don’t like those places and I’m screwed but I don’t like them. The less I go the better, you know”.

These responses were given by patients that were no longer at the psychiatric center while participating in the program. This fact also allowed them to be closer to their homes rather than have to travel the distance to the psychiatric center: “in my case, I would have to come from the sheltered housing to the psychiatric hospital every day by bus; it’s much further away” (Thomas).

Nevertheless, in some cases, some participants affirmed that they would have also participated if the program had been conducted in the psychiatric hospital. They also stated that the out-of-hospital program did not have as much influence when deciding to participate in it, since their addresses were in another community: “I tell you that I live in Logroño. There will be more people who have more difficulties. I don’t know the truth. I wouldn’t care” (Charles).

#### 3.1.2. Benefits of the Program: Level of Participants’ Satisfaction

Several aspects were highlighted by the participants during the conduction of the program, since it was beneficial in different aspects. First, the seriousness attributed to its implementation in sports facilities outside the hospital, as expressed by Reece: “when you come here to Mendizorroza, it’s sort of more serious, you know? You know what I mean? And you’re going to do it there in the hospital, it doesn’t seem funny, you know what I’m telling you, but it’s different. You see it and with this I make pam, pam. I don’t know what, but it’s not the same as coming here. You know you take it more seriously now”.

In addition, as the participants affirmed, the sports facilities had more means to guarantee more attractive physical practice adapted to their needs. At the same time, having more resources was also a determining factor for the satisfaction associated with the participation of the participants and the importance attributed to the program: “realize that this is IVEF (Faculty of Education and Sport), this is a specialized center; it is a sports university or whatever; this is not the same as a center… it doesn’t have the same name, the same incentive, you know!” (Noah), and “I liked it better in Mendizorroza, because it was more like a cool gym, and I don’t know, you went there, with your own showers, history” (Reece).

Other participants stated that leaving the hospital was very beneficial for individuals with SP. Therefore, differentiating the admission hospital and attending an out-of-hospital program helped create links with the outside world, which was a positive aspect according to the participants: “I think that if this continues this way, it would be very good for the individuals in there” (Max), and “being in the hospital I think it is very good to get out a bit and exercise” (Alexander).

The continuation of the program and the need to continue promoting this type of proposal was highlighted by the majority of the participants. They perceived themselves benefited in different aspects thanks to their participation, showing the desire that the program had a long life: “it would have to be extended as if it were a municipal sports gym that would be prolonged in time” (Charles).

Finally, there were those who stated that they wanted to do it again if that was possible. They felt very satisfied by having participated in the program, despite the fact that they initially did it for an altruistic cause such as helping by being part of the study: “ah! I would repeat it, if that’s what you mean. At first, it was to help the study, but I participated with pleasure during the program; I even had fun. It was good, a very good experience, come on, with the colleagues it was also good; we were all good; we didn’t have any conflicts between us, that is, there were no complaints” (Alexander).

### 3.2. Personnel Responsible for the Program: Qualified Exercise Professionals

#### 3.2.1. Qualified Personnel and Different from the Social and Healthcare Personnel

Practicing physical exercise with individuals specifically qualified in the field of physical activity and sports sciences was an aspect highlighted by the interviewees, since having personnel that did not come from the social and healthcare field―such as assistants, psychiatrists, or nurses―was valued by the majority. They emphasized that, without these professionals, they would not have been involved in the program and it would not have had the same approach: “with the auxiliaries and that can’t be done there, not at all, not at all, not at all, no, no” (Andrew), “no, no, no, neither. I don’t want anything. I don’t like it. I don’t like it. Psychiatrists and that stuff. No, no, no, no, no, no, no, I don’t like it” (Liam), and “I don’t know, I have an experience with you, and I think you did well, I think you also knew more about sports than nurses, right? I say that each one with respective functions” (Alexander).

There were those who stated having had tense relationships with the staff of the psychiatric center: “uh, well it’s going to be worse, huh. I don’t see much of a future for attending the project with the personnel of the psychiatric hospital. I see it more as a tug-of-war, because for me the psychiatric hospital has been a tug-of-war, one tells you one thing, the other tells you another and the other another and all they do is make you dizzy, each one has their way of thinking and all they do is make you dizzy” (William).

#### 3.2.2. Social and Relational Skills

Regarding the closeness and bond created with the individuals in charge of the program, the participants agreed that the teachers were always attentive to their needs, the treatment was very pleasant, and this fact made them more motivated to make an effort. They positively valued the teachers’ behaviors, sharing positive memories related to the experience: “well, I prefer that the monitors had been there. Very nice, very friendly, and very nice” (Kyle), “they are nicer, more apprehensive, they care more, more affectionate. More cuddling and things like that (than the hospital staff)” (Amelia), and “yes, yes, yes, I have good memories of the exercise, yes, yes, yes, and also the responsible personnel were very friendly and very attentive, and always aware of whether or not something hurt, yes, yes, yes, always attentive” (James).

There were those who highlighted that their experiences had been even more positive thanks to the qualities of the qualified exercise professionals, since it was a pleasant surprise for the participants and had effects in increasing their level of acceptance of the program: “I did not expect this; I did not expect the monitors as they were. I don’t know, I expected something else, well, more rigidity. […] yes, more rigid, and when I came across this, I accepted the program more” (Emily).

#### 3.2.3. Scheduled and Designed Sessions

On the one hand, the scheduling of the sessions and the way they were performed was positively valued by all the participants, highlighting their variability, as explained by William: “it is better designed, because they give us tasks and force us to do things, new things ehh… (which you don’t like doing on your own very much) no, I get more diverted, and I say again ‘one day I come and another day I don’t come’ and that is the problem”.

In addition, as in the previous statement, performing scheduled activities helped the participants persevere and not give up while trying to perform the activities. This way, the activities promoted some adherence to physical exercises. Therefore, there were those who related having performed designed activities with less boredom, as in the case of William: “those from COTA (Counseling and addiction treatment center) were good, but do you know what happens? That it’s good because they form a group and perform activities all together, they tell us to run, they tell us to do things, but then, once you go to the gym alone, you don’t go with monitors who tell us to perform activities and so on, they say ‘don’t come basketball, play basketball’ and we play basketball or you go to the machines, but it’s not like… (it’s not designed) it’s not designed exactly, and that’s more boring”.

The professionalism of the qualified exercise professionals was evident in the experiences related by the interviewees, both when it came to scheduling sessions in accordance to the needs of the users and when trying to offer individualized services to each participant as far as possible.

#### 3.2.4. Level of Involvement and Follow-Up 

The participants of the CORTEX-SP program valued very positively the follow-up provided by the professionals and how it influenced them in increasing their motivation for being more involved in the program, as highlighted in the words of different interviewees: “well, I don’t know what I can tell you, I’m doing very well. You are giving them hope and we also have to consider the patients, the individuals who participate. Yes, illusion and such things. Maybe I don’t know because of X, I lacked that motivation, I don’t know, I had it at one point, but well, other times I didn’t, but it’s ok” (Robert), and “with great desire and not only for the program, the young man you have there, the entertainer, he is worth a lot, because he’s all the time cheering you up; it seems that he increases your strength when you don’t have it. (The young man who comes to the hospital encourages me). He is very human and generates a lot of positivity, joy, and everything, you start walking, it seems that you can’t and you can do it more (laughs)” (Poppy).

The follow-up provided for continuing in the program was also valued very positively by a relative of a patient. This individual stated that the patient had never before achieved that level of involvement and commitment, showing gratitude for the progress of the relative in this adherence to physical exercise thanks to the qualified exercise professionals: “indeed, you know, that is, a very important part of the study is the daily follow-up, because she… you know, they obtained a commitment from her to come to the study that I had not achieved, you know, and the daily monitoring of ‘I’m not going today.’ Phone call, blah, blah, blah…, what’s up? How are you doing? Not today. Well, come on, see if you’ll come tomorrow; Joe called me, and their daily follow-up is the best part of the program. In other words, I have seen that he has managed to get something out of her, it has been them. (…) The commitment obtained by Michael and his partner, whose name I don’t remember exactly, is priceless” (Isla).

## 4. Discussion

Several qualitative [7,33,34] and quantitative [33,34,35,36,37] studies have determined the benefits and challenges related to the practice of physical exercise faced by individuals with SP. Physical exercise has been considered a good treatment for the disease [27]. In turn, it has been concluded that out-of-hospital programs, designed and supervised by qualified exercise professionals, lead to greater acceptance rates and lower dropout rates [20,23].

On the one hand, the out-of-hospital nature of the physical exercise program was highly valued by the participants of the present study, since it could result in greater optimism and well-being [38,39]. Additionally, they also highlighted the importance of these programs for escaping, thus allowing individuals with SP to perform activities outside the usual spaces [40]. This fact could be a first step before adhering to structured leisure activities or other daily activities outside the home. In the present study, all the participants highlighted the importance of differentiating the program from the psychiatric center through its location, becoming an external and liberating space [18].

Regarding the feelings generated by the out-of-hospital program, the interviewees associated sadness and negative feelings with the hospital center and, on the contrary, declared that the change of scenery had generated greater positivity and greater benefits for them. This result is in line with other findings that observed that physical exercise improved feelings of depression, anxiety, and negative symptoms [41]. In addition, it has been demonstrated that conducting these programs close to the patients’ homes―as in the case of the participants of the present study who were no longer admitted to the psychiatric hospital―was more beneficial and motivating due to the characteristics of the physical environment and the preferences of the patients with mental illnesses [42].

On the other hand, several aspects of the out-of-hospital program were beneficial for the participants. Firstly, they highlighted the seriousness assigned to the intervention, which could be related to a decrease in unsafe, stressful, and tense situations experienced by the participants [18]. Secondly, having more resources specific to the field of physical activity was also highly valued by the participants. It is worth mentioning that other studies had highlighted the negative consequences of having limited spaces [43], the specific restrictions of the premises [18], or the shortage of personnel [44]. Thirdly, the creation of a link with the outside world was also pointed out by some participants, a situation also highlighted in another study [40]. These authors concluded that only the fact of going out and wanting to interact with the outside was important for individuals with SP, since it could enable them to have more options to participate in leisure activities. Finally, the interviewees highlighted the desire to prolong and/or repeat the physical exercise program. They were aware of the positive nature of participating in it and the improvements obtained in their daily lives, which were associated with maintaining physical activity related to the three fundamental needs that include relationship, autonomy, and competence [45], thus increasing motivation and well-being.

The qualified professionals in charge of designing and supervising the physical exercise program―i.e., the physical-sports education professionals―were very important for the participants, since the fact of being personnel outside their usual social and healthcare field (psychiatrists or psychologists) and having specific training in the field of physical-sports activities was very successful. The same result was obtained in other studies, which found that the dropout rates of the programs were lower when they were designed by qualified professionals [46]. At the same time, it was found that these programs increased patient adherence to physical exercise activities [47].

Regarding the skills of these professionals highlighted by the interviewees, the most positive were associated with the open, close, and friendly nature of the professionals. In turn, these characteristics contributed to greater motivation of the participants, greater involvement with respect to the program, and a more relaxed and pleasant atmosphere, as observed in other programs with similar characteristics [15,18,42,48]. Furthermore, adding the out-of-hospital feature of the intervention to all of the abovementioned further boosted the benefits associated with physical exercise and significantly increased the participants’ general well-being. Their stigma was reduced, giving them the opportunity to lead as normal a life as possible thanks to these positive social interactions [40].

Participating in a programmed intervention, designed, and supervised by qualified professionals who were in charge of personalizing and adapting the activities to the characteristics, as well as the needs and interests of the participants, turned out to be very positive for them, as all the participants highlighted. This fact gives a more central role to qualified exercise professionals since they become the main axis of the program for the direction of physical exercise [49]. Furthermore, ensuring a properly scheduled and designed program prevents boredom and provides a structure that contributes to escaping from the daily or weekly routine [18], as observed in the present study. Also, these programs favor more exercise-focused support for individuals who might have more difficulty sticking to it [42]. Lastly, the level of involvement of the qualified professionals and the follow-up they provided to the participants resulted in better behaviors of the participants towards the exercises, educating them about the biopsychosocial benefits of physical activity [18]. In addition, this fact might result in a higher level of commitment of individuals with SP both in the programs and in their daily activities, as confirmed in the positive evaluation of the husband of one of the participants. He was even surprised by the level of commitment achieved by her wife thanks to the involvement and daily follow-up provided by the qualified exercise professionals. The participant felt valued and encouraged in her daily life. This way, she would not give up attempting and would continue in the program for all the benefits that participating in a physical exercise program of these characteristics brought her.

## 5. Conclusions

The participants of the present study highlighted two main characteristics of the CORTEX-SP program. On the one hand, the importance of the program being conducted out-of-hospital, and on the other hand, the fact that the individuals responsible for the program were qualified exercise professionals. They also highlighted the importance of conducting the program outside the psychiatric center. This fact led them to a higher level of satisfaction while the location provided them with greater seriousness and greater number of resources, and encouraged them to want to repeat the program or extend it. The personnel in charge of the program, i.e., the qualified exercise professionals, were also fundamental for the success of the program. The participants emphasized the qualifications of the personnel crucial for this type of program, their social skills, and the level of involvement with them, and the follow-up provided. They also highlighted the fact that the activities were programmed, designed, and supervised. These results are fundamental for the future implementation of physical exercise programs and the achievement of success for individuals diagnosed with SP. These programs will be vital for the prevention and treatment of cognitive dysfunctions, as well as the positive and negative symptoms caused by SP, aiming to help improve the quality of life of individuals affected by this pathology.

## Figures and Tables

**Table 1:** System of categories for data analysis.

Category	Subcategory	Code
Out-of-hospital program	Preference over the psychiatric center and influence on the level of participation	Escape enjoying a better environment
		Improvement of feelings: mood, sadness
		Mobility—proximity/distance from home
		Out-of-hospital participation
	Benefits of the program: level of participants’ satisfaction	Seriousness
		Available resources
		Link with the outside
		Intention to repeat—prolong the program
Individuals responsible for the program	Qualified exercise professionals	Qualified personnel and different from the social and healthcare personnel
		Social and relational skills
		Scheduled and designed sessions
		Level of involvement and follow-up

## Data Availability

Data are available upon request from the corresponding author.

## References

[1] Crawford P., Go K.V. (2022). Schizophrenia. Am. Fam. Physician.

[2] Rastad C., Martin C., Asenlöf P. (2014). Barriers, benefits, and strategies for physical activity in patients with schizophrenia. Phys. Ther..

[3] Ma H., Cheng N., Zhang C. (2022). Schizophrenia and Alarmins. Medicina.

[4] Lieberman J.A., First M.B. (2018). Psychotic Disorders. N. Engl. J. Med..

[5] Orzelska-Górka J., Mikulska J., Wiszniewska A., Biała G. (2022). New Atypical Antipsychotics in the Treatment of Schizophrenia and Depression. Int. J. Mol. Sci..

[6] American Psychiatric Association (2013). Diagnostic and Statistical Manual of Mental Disorders: DSM-5.

[7] Roberts S.H., Bailey J.E. (2013). An ethnographic study of the incentives and barriers to lifestyle interventions for people with severe mental illness. J. Adv. Nurs..

[8] Mestdagh A., Hansen B. (2014). Stigma in patients with schizophrenia receiving community mental health care: A review of qualitative studies. Soc. Psychiatry Psychiatr. Epidemiol..

[9] Laliberte-Rudman D., Yu B., Scott E., Pajouhandeh P. (2000). Exploration of the perspectives of persons with schizophrenia regarding quality of life. Am. J. Occup. Ther..

[10] Ciudad A., Bobes García J., Álvarez E., San Molina L., Novick D., Gilaberte Asín I. (2011). Resultados Clínicos Relevantes en Esquizofrenia: Remisión y Recuperación. Rev. De Psiquiatr. Y Salud Ment..

[11] Bueno-Antequera J., Munguía-Izquierdo D. (2020). Exercise and Schizophrenia. Adv. Exp. Med. Biol..

[12] International Advisory Group for the Revision of ICD-10 Mental and Behavioural Disorders (2011). A conceptual Framework for the Revision of the ICD-10 Classification of Mental and Behavioural Disorders. World Psychiatry.

[13] Miyamoto S., Miyake N., Jarskog L.F., Fleischhacker W.W., Lieberman J.A. (2012). Pharmacological treatment of schizophrenia: A critical review of the pharmacology and clinical effects of current and future therapeutic agents. Mol. Psychiatry.

[14] Callaghan P. (2004). Exercise: A neglected intervention in mental health care?. J. Psychiatr. Ment. Health Nurs..

[15] Crone D., Guy H. (2008). ‘I know it is only exercise, but to me it is something that keeps me going’: A qualitative approach to understanding mental health service users’ experiences of sports therapy. Int. J. Ment. Health Nurs..

[16] Stubbs B., Vancampfort D., Hallgren M., Firth J., Veronese N., Solmi M., Brand S., Cordes J., Malchow B., Gerber M. (2018). EPA guidance on physical activity as a treatment for severe mental illness: A meta-review of the evidence and Position Statement from the European Psychiatric Association (EPA), supported by the International Organization of Physical Therapists in Mental Health (IOPTMH). Eur. Psychiatry.

[17] Tous-Espelosin M., de Azua S.R., Iriarte-Yoller N., MartinezAguirre-Betolaza A., Sanchez P.M., Corres P., Arratibel-Imaz I., Sampedro A., Pena J., Maldonado-Martin S. (2021). Clinical, physical, physiological, and cardiovascular risk patterns of adults with schizophrenia: CORTEX-SP study: Characterization of adults with schizophrenia. Psychiatry Res..

[18] Soundy A., Stubbs B., Probst M., Hemmings L., Vancampfort D. (2014). Barriers to and Facilitators of Physical Activity among Persons with Schizophrenia: A Survey of Physical Therapists. Psychiatr. Serv..

[19] Karlsson V., Danielsson L. (2020). Motivators for patients with schizophrenia spectrum disorders to start and maintain exercising: A qualitative interview study. Eur. J. Physiother..

[20] Vancampfort D., Sánchez C.P.R., Hallgren M., Schuch F., Firth J., Rosenbaum S., Van Damme T., Stubbs B. (2021). Dropout from exercise randomized controlled trials among people with anxiety and stress-related disorders: A meta-analysis and meta-regression. J. Affect. Disord..

[21] Brand S., Colledge F., Beeler N., Pühse U., Kalak N., Sadeghi Bahmani D., Mikoteit T., Holsboer-Trachsler E., Gerber M. (2016). The current state of physical activity and exercise programs in German-speaking, Swiss psychiatric hospitals: Results from a brief online survey. Neuropsychiatr. Dis. Treat..

[22] Johnstone R., Nicol K., Donaghy M., Lawrie S. (2009). Barriers to uptake of physical activity in community-based patients with schizophrenia. J. Ment. Health.

[23] Gomes E., Bastos T., Probst M., Ribeiro J.C., Silva G., Corredeira R. (2014). Effects of a group physical activity program on physical fitness and quality of life in individuals with schizophrenia. Ment. Health Phys. Act..

[24] Ruiz Olabuénaga J.I. (2012). Metodología de la Investigación Cualitativa.

[25] Guerrero L.M. (2001). La Entrevista en el Método Cualitativo.

[26] Eatough V., Smith J.A. (2008). Interpretative Phenomenological Analysis.

[27] Tous-Espelosin M., Crone D., Ruiz de Azua S., Iriarte-Yoller N., Sampedro A., Maldonado-Martín S. (2023). ‘It Helped Me to Disconnect My Mind from the Problems’: The Subjective Experiences of People with Schizophrenia Taking Part in a Concurrent Exercise Program. Issues Ment. Health Nurs..

[28] Rapley T. (2014). Los Análisis de la Conversación, del Discurso y de Documentos en Investigación Cualitativa.

[29] Braun V., Clarke V. (2006). Using thematic analysis in psychology. Qual. Res. Psychol..

[30] Thomas J., Harden A. (2008). Methods for the thematic synthesis of qualitative research in systematic reviews. BMC Med. Res. Methodol..

[31] O’Connor C., Joffe H. (2020). Intercoder Reliability in Qualitative Research: Debates and Practical Guidelines. Int. J. Qual. Methods.

[32] Schmidt C., Flick U., von Kardoff E., Steinke I. (2004). The Analysis of Semi-Structured Interviews. A Companion to Qualitative Research.

[33] Chalfoun C., Karelis A., Stip E., Abdel-Baki A. (2015). Running for your life: A review of physical activity and cardiovascular disease risk reduction in individuals with schizophrenia. J. Sports Sci..

[34] Wu M.H., Lee C.P., Hsu S.C., Chang C.M., Chen C.Y. (2015). Effectiveness of high-intensity interval training on the mental and physical health of people with chronic schizophrenia. Neuropsychiatr Dis. Treat..

[35] Andersen E., Bang-Kittilsen G., Bigseth T.T., Egeland J., Holmen T.L., Martinsen E.W., Stensrud T., Engh J.A. (2020). Effect of high-intensity interval training on cardiorespiratory fitness, physical activity and body composition in people with schizophrenia: A randomized controlled trial. BMC Psychiatry.

[36] Bang-Kittilsen G., Engh J.A., Holst R., Holmen T.L., Bigseth T.T., Andersen E., Mordal J., Egeland J. (2022). High-intensity interval training may reduce depressive symptoms in individuals with schizophrenia, putatively through improved VO_2_max: A randomized controlled trial. Front. Psychiatry.

[37] Martland R., Stubbs B. (2019). High-intensity interval training: An adjunctive treatment for schizophrenia spectrum disorders?. Acta Psychiatr. Scand..

[38] Warner R. (2009). Recovery from schizophrenia and the recovery model. Curr. Opin. Psychiatry.

[39] Karow A., Moritz S., Lambert M., Schöttle D., Naber D. (2012). Remitted but still impaired? Symptomatic versus functional remission in patients with schizophrenia. Eur. Psychiatry.

[40] Lloyd J., Lloyd H., Fitzpatrick R., Peters M. (2017). Treatment outcomes in schizophrenia: Qualitative study of the views of family carers. BMC Psychiatry.

[41] Ho P.A., Dahle D.N., Noordsy D.L. (2018). Why Do People With Schizophrenia Exercise? A Mixed Methods Analysis Among Community Dwelling Regular Exercisers. Front. Psychiatry.

[42] Chapman J.J., Fraser S.J., Brown W.J., Burton N.W. (2016). Physical activity preferences, motivators, barriers and attitudes of adults with mental illness. J. Ment. Health.

[43] Gorczynski P., Faulkner G., Cohn T. (2013). Dissecting the Obesogenic Environment of a Psychiatric Setting: Client Perspectives. Can. J. Community Ment. Health.

[44] Happell B., Dares G., Russell A., Cokell S., Platania-Phung C., Gaskin C.J. (2012). The relationships between attitudes toward seclusion and levels of burnout, staff satisfaction, and therapeutic optimism in a district health service. Issues Ment. Health Nurs..

[45] Vancampfort D., Probst M., Scheewe T., De Herdt A., Sweers K., Knapen J., van Winkel R., De Hert M. (2013). Relationships between physical fitness, physical activity, smoking and metabolic and mental health parameters in people with schizophrenia. Psychiatry Res..

[46] Stubbs B., Firth J., Berry A., Schuch F.B., Rosenbaum S., Gaughran F., Veronesse N., Williams J., Craig T., Yung A.R. (2016). How much physical activity do people with schizophrenia engage in? A systematic review, comparative meta-analysis and meta-regression. Schizophr. Res..

[47] Vancampfort D., Rosenbaum S., Probst M., Connaughton J., du Plessis C., Yamamoto T., Stubbs B. (2016). What are the top 10 physical activity research questions in schizophrenia?. Disabil. Rehabil..

[48] Fogarty M., Happell B. (2005). Exploring the benefits of an exercise program for people with schizophrenia: A qualitative study. Issues Ment. Health Nurs..

[49] Stubbs B., Soundy A., Probst M., De Hert M., De Herdt A., Vancampfort D. (2014). Understanding the role of physiotherapists in schizophrenia: An international perspective from members of the International Organisation of Physical Therapists in Mental Health (IOPTMH). J. Ment. Health..

